# Southern Medical Reports

**Published:** 1849-08

**Authors:** E. D. Fenner

**Affiliations:** New Orleans, La.


It affords us pleasure to give place to the following prospectus. Dr. Fenner is eminently qualified for the undertaking which he proposes, and his efforts in behalf of American medical literature should be appreciated and encouraged.

The undersigned proposes to publish an annual volume, on the meteorology, medical topography, and diseases of the Southern States.

SOUTHERN MEDICAL REPORTS.

The object of this work, is to collect and present, in a *durable form*, the observations of physicians residing in different parts of the Southern States, with the view to the cultivation of medical science, and the formation of the medical history of the times.

It will consist of GENERAL and SPECIAL REPORTS; the first to contain concise accounts of the *meterology, medical topography and prevailing diseases* throughout the year; the second will be devoted to *extraordinary cases, surgical operations, &c.*

These reports are all to be handed to the Editor, by the first of January, and will appear in a *neatly bound volume*, as soon thereafter as the work can be done. Each volume will also contain a brief retrospect of the latest discoveries and improvements contained in the Medical Journals of the year.

The Editor wishes it to be distinctly understood, that this work is not designed to conflict with the Medical Journals of the South. On the contrary, he hopes his collaborators will contribute all they can to their encouragement and support. If the Editor can command the co-operation of his professional brethren, each volume of this work will contain reports from prominent points in the following States, viz: North and South Carolina, Georgia, Florida, Louisiana, Texas, Alabama, Mississippi, Arkansas, and Tennessee. Also from the southern stations of the U. S. Army and navy. Special reports of interesting cases will be thankfully received from any part of the South. The cost of this work will be in proportion to the extent of patronage. It is contemplated that each volume will contain from *five to six hundred pages*, and will be furnished to subscribers at $3 50. One copy will be sent gratuitously to all contributors, and three or more copies to the authors of annual reports.

All Medical Societies within the above limits, are respectfully invited to send in condensed reports of their transactions during each year.

Publishers are requested to forward copies of all new medical books; in return for which, bibliographical notices will be given.

Medical Colleges throughout the Union, are requested to forward their annual circulars; from which will be extracted statistics, to show the progress of medical education. In short, the proposed work is designed to make up the medical history of the time, and to promote the cultivation of medical science in the Southern States. The Editor will do all in his power to render the work advantageous, as well to the collaborators as to the profession at large.

E. D. FENNER, M. D.,

*New Orleans, La., June* 1, 1849. *No.* 5, *Carondelet Street.*

☞ Due notice will be given when the work is completed, and it will be deposited at the principal commercial points in the Union.

